# What Ails the Dental Societies?

**Published:** 1881-12

**Authors:** 


### WHAT AILS THE DENTAL SOCIETIES?

*Editor of the Register—Dear Sir*:

Through the kindness of the Ohio State Dental Society I have received a copy of the printed transactions for 1880 — the fifteenth annual meeting. The Society is certainly doing good missionary work in sending its discussions to all who apply, and many who do not, in all parts of the world.

Some of its members complain that the meetings are not as well attended as they should be, and, excepting the faithful few, those present evince little or no interest in the work. Out of the 700 or more dentists in the State only about 100 are connected with the Society.

Now, Mr. Editor, I do not pretend to know "the why and wherefore" of this state of affairs, but was, "once upon a time," a member of a dental society which seemed to suffer in the same manner as the Ohio Society.

The average member expected great things to be done, although *he* did not feel bound to do anything. The dentist who did not attend the meetings or help to sustain the society expected even more than the average member. He had known the list of subjects for discussion at the annual meeting during a whole year, yet had not given it a thought or noted a fact that would be of use in the discussions. He generously left that to some one else, ("Some fellows run it every year, any way, you know,") and some one else left it to—well, to chance.

So did the officers and the committees. At the eleventh hour the executive committee rushed off to a printing office and got out an announcement of the meeting--so had their predecessors. During the previous twelve months nothing had been done to make the coming session more interesting or different from the last one. No papers to announce, no clinics, no lectures or exhibition, absolutely nothing except the bare "list of subjects for discussion." They had done precisely as the committees before them.

True, no one had offered to do any of these things; equally true that they had not asked any one. If thé material could not be found in one state it might have been found in another, and there was a whole year in which to secure it. The members could at least have kept the list of subjects in mind, and at the meeting, if they could not present a paper or carry on the discussion, ask questions of their more gifted brethren.

The before mentioned average member, however, treated the meeting as a side-show attached to the dental depots--spending his days at the latter and his evenings at the theater or in other amusements. He looks in at the meeting occasionally, being always careful to secure a seat near the door, and, perhaps, sees a "bore-with-me" member putting to sleep all those within reach of his voice, the others are intrenched behind newspapers.

This b. w. m. member usually arrives late, just in time to catch the last words of a discussion. Some one moves that the subject be passed. It brings the b. w. m. member to his feet. He objects, most decidedly, to its being passed in that hasty manner; there are several things that might be said on the subject, etc., etc.

The chairman informs him that the points he mentions were thoroughly discussed before his arrival; but, paying no attention to the chairman, be begins at the very beginning of things, and, neither eloquent nor learned, he soon makes everything "as clear as mud." After twenty or thirty minutes of monotonous speech he complacently observes, "Now, Mr. President, I have no objection to the consideration of the next subject," and sits down with a satisfied air.

Should any one attempt to regulate the b. w. m. member, he looks around, says he wants but a few minutes, (to begin, I suppose,) and the good natured members allow him to proceed. He repeats the same performance year after year, becomes confirmed in the habit, and never has anything new to communicate.

The average member soon "spots" and, whenever he commences to talk, strikes a bee-line for the more congenial dental depot.

Then there is the inspired member. Though he may think and have ideas, it is evident nature never intended him to deliver them. After a persistent appeal, with his eyes, to the ceiling and an heroic struggle, he gets through.

Now the funny member must "have a lick at it" as he says. There is a total lack of science in his talk, but he is fluent of speech, and the sleepers awaken, newspapers are laid aside, and all listen to his sharp thrusts. Is it any wonder that we envy that average member at the dental depot over the way?

"O, wad some power the giftie gie us
To see oursel's as others see us!
It wad frae monie a blunder free us
And foolish notion."

Besides the time wasted in listening to those who had nothing to say, there was the personal wrangles of a few over the miscellaneous business of the meeting. A remedy would be in committees to whom allsuch matters should be referred for settlement. Members wishing to discuss anything so referred, could go before the proper committee, and not consume the time needed for the consideration of the regular topics.

If the reforms, which will suggest themselves, could be carried out, it seems to me that greater interest would be felt in our societies, and all do better work for the cause. KALAMAZOO.

